# Flexible word position coding in reading: Roles for attention and memory

**DOI:** 10.3758/s13421-024-01623-7

**Published:** 2024-08-22

**Authors:** Joshua Snell

**Affiliations:** Experimental and Applied Psychology, Vrije Universiteit Amsterdam, Amsterdam, The Netherlands

**Keywords:** Reading, Word position coding, Attention, Memory

## Abstract

Readers may fail to notice the error in '*Do love you me?*': this is the recently established transposed-word (TW) phenomenon. Word position coding is a novel cognitive construct, and researchers are presently debating the underlying mechanisms. Here I investigated roles for attention and memory. Participants (N = 54) made grammaticality judgements to four-word sequences that formed correct sentences ('*The man can run*', '*The dog was here*'), TW sentences ('*The can man run*', '*The was dog here*'), or ungrammatical control sentences ('*The man dog run*', '*The was can here*'). Sequences were replaced by post-masks after 200 ms, and that post-mask was accompanied by a 50-ms retro-cue in the form of an 'X' presented at a critical location (where one could have locally inferred grammaticality; e.g., between the first and second word of '*The was dog here*') or a non-critical location (e.g., between the third and fourth word of '*The was dog here*'). TW sentences were harder to reject than control sentences – the classic TW effect – and crucially, this effect was modulated by cue validity, with valid cues attenuating TW effects compared to invalid cues. The present results suggest that focused attention aids the process of binding words to locations. Furthermore, as cues appeared after sentence offset, these results suggest that word position coding may take place in memory.

## Introduction

How do you see the difference between '*baby dog eats meat*' and '*baby eats dog meat*'? Knowledge of word order likely depends on multiple things. Vision may provide the most straightforward contribution: if the letters from '*dog*' and '*eats*' are situated in different respective hemifields, then this perceptual information alone should already inform the relative positions of '*dog*' and '*eats*' to some degree (e.g., Snell, 2024). Visuo-spatial attention and oculomotor strategies likely contribute as well: by moving attention and the eyes from left to right, focusing on words one-by-one, there is a good chance that words are perceived in their printed order.^1^ Additionally researchers have hypothesized a role for top-down predictions, such as knowledge of the grammatical rules of a language, which would, for instance, make one more likely to perceive a verb at position 3 when one has recognized a noun at position 2, and vice versa (e.g., Gibson, Bergen, & Piantadosi, 2013; Snell, van Leipsig, Grainger, & Meeter, 2018).

The various contributors to knowledge of word order do not always amount to an accurate percept. Instead, recent studies suggest that this knowledge is at times incomplete, uncertain and malleable. This is most clearly illustrated by the '*Do love you me?*' phenomenon – more formally known as the transposed word (TW) phenomenon – whereby readers tend to fail to notice the incorrect positions of '*you*' and '*love*'. Experimentally this is revealed in a grammaticality judgement task, where the typical finding is that it is more difficult to detect the ungrammaticality of TW sentences (e.g. '*The can man run*', '*The was dog here*') than of ungrammatical sentences that are always incorrect irrespective of the perceived order of words (e.g., '*The man dog run*', '*The was can here*') (e.g., Hossain & White, 2023; Huang & Staub, 2021, 2023; Liu et al., 2021, 2022; Milledge et al., 2023; Mirault et al., 2018, 2022; Snell & Grainger, 2019; Snell & Nogueira-Melo, 2024; Snell et al., 2023).

These recent investigations have led to the general understanding that, given a sufficient degree of flexibility, the brain ties words to grammatically expected locations. But what gives way to that flexibility in the first place? And what mechanism ties words to locations? These are open problems. In the present paper I report an experiment that reveals roles for memory and attention in word position coding, informing both issues at once. In the remainder of this *Introduction* I firstly reflect on the determinants of flexibility (see the Section *A role for memory*), highlighting current debates which are largely focused on whether flexibility occurs in a perceptual or rather post-perceptual (memory) stage. Subsequently I hypothesize a role for focused attention in the process of mapping words onto locations (see the Section *A role for attention*), drawing inspiration from Feature Integration Theory (Treisman, 1977; Treisman & Gelade, 1980).

### A role for memory

There are currently several competing accounts of TW effects. Some have argued that flexibility in word position coding is induced by a widespread distribution of attention, allowing parallel processing of multiple words and, consequentially, potential confusion about word order (e.g., Mirault et al., 2018, 2022; Snell & Grainger, 2019; Snell & Nogueira-Melo, 2024). According to this scenario grammatical expectations would operate early, impacting at once word recognition and word position coding during perceptual stages of processing. An alternative explanation of TW effects is that word recognition is a strictly serial process (i.e., attention is directed to one word at a time) – meaning words are recognized in their printed order – but words may subsequently be rearranged in a post-lexical buffer in working memory. According to this scenario, flexibility in word position coding, and the consequential impact of grammatical expectations on perceived word order, would occur relatively late (e.g., Hossain & White, 2023; Huang & Staub, 2021, 2023; Liu et al., 2022; Milledge et al., 2023; and for similar accounts of flexibility in spoken language comprehension, see, e.g., Gibson et al., 2013). And yet another scenario is one where word position coding happens both during and after perception. After all, widespread attention and a post-lexical buffer are not mutually exclusive.

Does current evidence allow us to adjudicate among the three scenarios? One particularly important variation of the TW paradigm has compared TW effects between a setting where all words are presented simultaneously and a setting where the same words are presented sequentially (e.g., Hossain & White, 2023; Huang & Staub, 2023; Liu et al., 2022; Milledge et al., 2023; Mirault et al., 2022; Snell & Nogueira-Melo, 2024). The general finding here is that TW effects can also occur in the latter condition; (the one exception being the study of Snell and Nogueira-Melo (2024) in which TW effects solely occurred in the parallel presentation condition). The fact that TW effects can in principle occur despite seeing words one by one suggests that widespread attention is not the sole determinant of flexibility in word position coding. Indeed, the fact that a currently viewed word may 'trade places' with a bygone word implies that, to some extent, memory plays a role too. On the other hand, in most of the aforementioned studies TW effects were decidedly stronger in parallel presentation; and Snell and Nogueira-Melo (2024) have argued that this difference cannot be accounted for by a post-lexical buffer alone.

On the basis of current evidence, one might posit that flexibility in position coding is induced during both perceptual and post-perceptual stages, whereby TW effects in serial presentation conditions point to an involvement of memory, while disproportionally stronger TW effects in parallel presentation conditions point to a concurrent role for widespread attention. It should be noted, however, that it is difficult to pinpoint the respective contributions of perceptual and post-perceptual processes. This is because various aspects of reading may play out quite differently between the two settings – in spite of good attempts to keep conditions as equal as possible. To illustrate, most aforementioned studies have matched the total presentation duration between serial and parallel presentation, meaning a four-word sequence would be shown for 1,000 ms in parallel presentation, and 250 ms per word in serial presentation. But participants typically respond earlier in parallel than serial presentation (e.g., Snell & Nogueira-Melo, 2024), and it is therefore possible that less time was spent on each word in parallel presentation than the 250-ms that participants were forced to spend on each word in serial presentation. Possibly the number of words that simultaneously occupy the post-lexical buffer is a function of reading speed, with faster reading leading to a greater number of words being considered simultaneously (see, e.g., Huang & Staub, 2023, for a similar reasoning). This may have given way to greater susceptibility to TW effects in parallel than serial presentation. Hence, taking stock of the evidence thus far, only the involvement of memory – as evidenced by TW effects in serial presentation – seems relatively certain at present. That is not to say that attention is unlikely to play an important role: indeed, it will be seen in due course that the results of the present study do implicate attention directly.

What might a representation of word order in memory look like? According to the OB1-reader model (Snell et al., 2018a, 2018b), the reading brain maintains a spatiotopic sentence-level representation in working memory, whereby text constitutes an array of 'blobs'. Each of these blobs has its own respective spatial coordinates, and each recognized word is mapped onto a blob, guided by low-level visual cues (e.g., perceived word lengths) and top-down grammatical constraints.

If word positions are indeed encoded via a spatiotopic representation in working memory, then this process may be probed in various ways. I elaborate on this below. First, however, let us consider what mechanism allows the brain to tie words to locations in this spatiotopic representation.

### A role for attention

I noted at the start of this *Introduction* that attention is a likely contributor to the inference of word order. The simplest reason for this would be that attention – in the most general sense conceptualized as the brain's ability to prioritize one source of information over another (e.g., Desimone & Duncan, 1995) – would offer the brain a way to recognize words sequentially, meaning words are ordered via separation in time. But this is not how knowledge of word order is conceptualized in OB1-reader. Instead, in OB1's spatiotopic representation, words are ordered via separation in *space*. However, it so happens that, outside the realm of reading, attention has long been implicated in spatial localization too.

Here I draw a parallel to Feature Integration Theory (Treisman & Gelade, 1980) and modern adaptations of it (e.g., Hulleman & Olivers, 2017; Reynolds & Desimone, 1999; Treisman, 2006; Wolfe & Horowitz, 2017) to hypothesize that focused attention is used to tie words to positions. Feature integration theory is traditionally offered as a solution to the so-called 'binding problem', which considers the fact that different visual features of an object (e.g., shape, color) are processed by different brain regions, meaning the brain must have some mechanism in place to link activity across multiple separate neural populations in order to perceive objects as a whole (e.g., Feldman, 2013; Golledge et al., 2003; Whitney, 2009). The theory champions visuo-spatial attention as the key mechanism: by directing attention to an object's location, the neurons that encode its constituent features receive a joint boost in activation, and this would allow the brain to deduce that those activated features belong to the same location and thus the same object.

Application of the above logic to the realm of reading begs but a small leap of the imagination. Undoubtedly word recognition warrants recognition and localization of various visual features, for example, detection of the letter 'T' relies on detection of a horizontal and a vertical line, and the brain's ability to localize these features should further allow it to know the difference between the letters 'T' and 'L'; (these processes are formalized in the seminal Interactive Activation Model of McClelland and Rumelhart (1981)). It deserves mentioning that several tests of Feature Integration Theory already made use of letter stimuli, showing that observers may mix up features between letters when not focusing attention on them (e.g., Butler et al., 1991). The rules that govern the interface between letters and their constituent features also apply to the interface between words and letters, with word recognition relying on the detection and localization of constituent letters (see, e.g. Grainger, 2008, for a review). Now, given evidence that the brain receives orthographic input from multiple words in parallel (e.g., Angele et al., 2013; Dare & Shillcock, 2013; Grainger et al., 2014; Snell et al., 2017; Snell et al., 2018a, 2018b; Snell et al., 2019), what prevents the brain from linking letters to incorrect word locations? Reports of so-called letter migration effects (e.g., '*land sane*' being read as '*sand lane*') show that this can in fact happen occasionally (e.g., Davis & Bowers, 2004; Vandendaele et al., 2019). Feature Integration Theory's answer would be that focused attention ties letters to correct word positions; and conversely, letter migration errors would result from a failure to focus attention on single words. Possibly, then, the transposed word effect is nothing but an exaggerated letter migration effect, involving not just two but all letters between two words. At any rate, TW effects would result from a failure to properly focus attention on single word locations.

But what about the supposed role for memory in word position coding, as motivated in *A role for memory*? Here it is important to consider that attention does not only operate in the external world during perception, but also internally for representations held in working memory. There is now a vast literature to suggest that the brain orients attention internally to prioritize retention of one item over another; and spatial attention in visual working memory appears to be quite analogous to spatial attention in visual perception (e.g., Awh et al., 2000, 2006; Downing, 2000; Nobre et al., 2004; Olivers & Roelfsema, 2020; Theeuwes et al., 2011). It has in fact been suggested that Feature Integration Theory's central cognitive process of interest – the process of binding features to locations – largely unfolds in visual working memory (e.g., Fougnie & Marois, 2009; Treisman & Zhang, 2006; Zokaei et al., 2014).

Now we have all the ingredients for a general hypothesis about word position coding. Following the OB1-reader model (Snell et al., 2018a, 2018b), readers' first glance at a sentence is thought to activate a spatiotopic representation that conveys the locations of to-be-recognized words in working memory. In the spirit of Feature Integration Theory (Treisman & Gelade, 1980), spatial attention is then thought to be directed at those locations in working memory in order to bind activated words to their respective positions. Note, I still leave open the possibility that this process partly plays out during perceptual stages of processing too. But the above scenario already generates important and testable predictions, as explained below.

### The present study

The present work was driven by the conception that, if word position coding relies on a spatiotopic representation in working memory (Snell et al., 2018a, 2018b), then this process can be probed in various ways. Specifically, because the spatiotopic representation is thought to be tied to the spatial layout of stimuli in the visual field, the prediction is that spatial cues can impact word position coding; and moreover, because the spatiotopic representation is maintained in working memory, those spatial cues should impact word position coding even if they occur *after* the text has disappeared.

A wealth of prior research has shown that visual working memory can be manipulated by offering retro-cues after stimulus offset, whereby the typical finding is that cueing spatial attention towards a bygone visual object's location leads to better retention of that object (e.g., Li & Saiki, 2014; Robison & Unsworth, 2017; Souza & Oberauer, 2016). Hence, cues in the external world can bias internal attention. In the present study I exploit this principle to see if retro-cueing attention to critical versus non-critical locations biases word position coding. The hypothesis is that retro-cues at critical locations (where one could have locally inferred grammaticality; e.g., the first two words of '*the was dog here*') would attenuate TW effects as compared to retro-cues at non-critical locations (e.g., the last two words of '*the was dog here*').

All stimuli, software, data and analysis code are openly accessible via the Opne Science Framework at https://osf.io/qx8c5/

## Methods

### Participants

Sixty-two volunteering students participated in this study for monetary compensation or course credit. All participants reported to be native to the Dutch language, non-dyslexic and to have normal vision. Only participants with an overall accuracy > 0.60 were retained for the analysis, which reduced the sample size to 54.

### Materials and design

The experiment was a 3 × 3 factorial design with Grammaticality (Grammatical, Ungrammatical TW, Ungrammatical control) and Cue (Valid, Invalid, No-cue) as factors. I employed the stimulus set of Snell and Nogueira-Melo (2024), which consisted of 80 Dutch four-word sentences (with an average word length of ~ 3.76 letters). Following several critical manipulations, each of these sentences occurred once in the Ungrammatical TW condition, once in the Ungrammatical Control condition, and twice in the Grammatical condition. The latter was to have an equal number of Grammatical versus Ungrammatical trials. The Grammatical trials were merely used to induce the task, and were not included in the analyses.

A TW version of each sentence was created by switching two adjacent words.^2^ Ungrammatical Control sentences were created by pairing grammatical sentences and switching two words within each pair. Cues would appear in between two words, whereby Valid Cue locations were defined as those locations where one could have locally – i.e., on the basis of the surrounding two words – inferred grammaticality, such as at ‘*the was*’ in ‘*the was dog here*’. Invalid Cue locations were locations where the local grammaticality did not align with the sequence grammaticality. For every base sequence, valid and invalid cue locations were matched between its TW and Ungrammatical Control counterparts. The same cue locations were used in the Grammatical condition too, even though there was technically no manipulation of cue validity in these trials; (after all, in a correct sentence, all cue locations would correctly signal grammatical soundness). Note, depending on the base sequence and on the manipulated words, there could be multiple possible valid or invalid cueing locations. Per sequence I chose the cueing locations in such a way that, across all sequences, there was a decent spread of cueing locations.^3^ Examples of all cueing conditions for TW and Ungrammatical Control sequences are provided in Table 1.

**Table 1:** Condition examples for two base sequences, whereby the ‘X’ indicates the location of the cue. The ‘X’ is presented in red here for the sake of visibility. It was presented in black in the actual experiment

	No cue	Valid cue	Invalid cue
Grammatical base 1	we should leave them	we should X leave them	we X should leave them
TW	we leave should them	we leave X should them	we X leave should them
UC	we dance leave them	we dance X leave them	we X dance leave them
Grammatical base 2	this dance works well	this dance X works well	this X dance works well
TW	this works dance well	this works X dance well	this X works dance well
UC	this should works well	this should X works well	this X should works well

*TW* transposed word, *UC* ungrammatical control

Using a Latin-square design with two groups I ensured that all sentences were shown in all conditions, but every individual participant saw each sentence only six times (once per Cue condition in the Grammatical condition, and once per Cue condition in either the Ungrammatical TW condition or the Ungrammatical Control condition). As such there were 40 measurements per condition per participant; and the resulting 2,160 measurements per condition exceeds the recommended 1,600 measurements for ~ 0.80 statistical power in LMM analyses (e.g., Brysbaert & Stevens, 2018). Words were presented in monotype font along the horizontal meridian, with each character subtending 0.3 degrees of visual angle. The total of 480 trials was presented in random order.

### Procedure

**Fig. 1:**
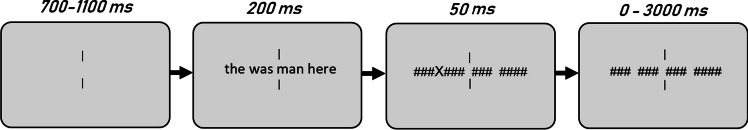
Trial procedure. The size of stimuli relative to the screen is exaggerated here for better visibility

The experiment was implemented with OpenSesame (Mathôt et al., 2012). A schematic overview of the trial procedure is shown in Fig. 1. Each trial started with central vertical fixation bars for 700—1,100 ms. Then the four words were shown for 200 ms. The four words were subsequently replaced by a mask stimulus that consisted of a hashmark at each previous letter location. In the Valid and Invalid Cue trials, the mask was shown during the first 50 ms accompanied by a cue in the form of an uppercase 'X' in between two words. The mask stayed onscreen until response. Participants were instructed to respond as quickly and accurately as possible with the '/' key for grammatical sentences and the 'Z' key for ungrammatical sentences. Feedback was provided with a central green dot for a correct response, or a red dot in case of an incorrect response or having reached a time-out of 3,000 ms. The experiment lasted approximately 25 min in total and participants were offered a break halfway through.

## Results

As noted before, eight out of 62 participants were excluded from the analyses due to having an overall accuracy < 0.60. For the remaining 54 participants, I retained only TW trials and Ungrammatical control trials. For analyses of response time (RT), I excluded incorrectly answered trials (~ 34%). For analyses of accuracy and RT, I further excluded trials with an RT 2.5 SD from the grand mean (1.3% and 1.2% of trials for analyses of accuracy and RT, respectively).

Data were analysed with linear mixed-effect models (LMMs) with Grammaticality (TW vs. Ungrammatical Control) and Cue (Valid vs. Invalid vs. No-cue) as fixed effects and Participants and Items as random effects. Models were computed in R (version 3.6.1) with the lme4 package (Bates et al., 2015). LMMs successfully converged with the maximal random effect structure, meaning models comprised by-participant and by-item random slopes for both fixed effects as well as their interaction.^4^ Accuracy was analysed with a generalized LMM, which also converged successfully with the maximal random effect structure. Alongside *b*-values and SEs, I report *t*-values or *z*-values for normal and generalized LMMs respectively, with values | *t* | and | *z* |> 1.96 deemed significant. To ensure that data were normally distributed, I applied a log-transformation prior to the analysis of RT. However, to aid interpretation of effects I plotted raw data in the graphs.

**Fig. 2:**
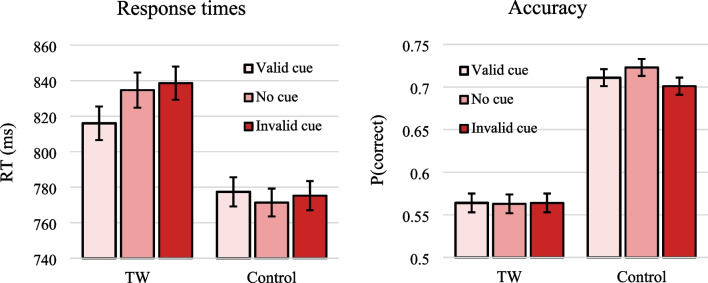
Mean response times and accuracies per condition. Error bars depict SEs

Results for RTs and accuracy are shown in Fig. 2. I replicated prior research with a TW effect in both RT and accuracy, whereby it was decidedly more difficult to reject TW sentences than Ungrammatical Control sentences (RT: *b* = 0.09, SE = 0.02, *t* = 5.87; accuracy: *b* = 0.61, SE = 0.11, *z* = 5.56). I also observed a main effect of Cue in RT, with faster responses for Valid compared to Invalid Cues (with Valid Cue as reference: *b* = 0.03, SE = 0.01, *t* = 2.27). In accuracy this main effect did not, however, reach significance (*b* = 0.10, SE = 0.07, *z* = 1.46). For the No-cue condition, performance was situated somewhat in between those of the Valid and Invalid cue trials, though the No-cue trials differed significantly from neither; (with No-cue as reference: in RTs, respectively, *b* = -0.02, SE = 0.02, *t* = -1.36 and *b* = 0.01, SE = 0.02, *t* = 0.76; in accuracy, respectively, *b* = 0.01, SE = 0.07, *z* = 0.09 and *b* = 0.11, SE = 0.07, *z* = 1.58).

Given the hypothesis that focused attention ties words to locations in working memory, the crucial test was whether TW effects would be modulated by Cue validity. This interaction (tested without inclusion of the No-cue trials) was established: in RT, TW effects were reduced upon retro-cueing attention to critical locations compared to non-critical locations (*b* = 0.04, SE = 0.02, *t* = 2.04). No significant interaction was observed in accuracy (*b* = 0.16, SE = 0.10, *z* = 1.57).

## Discussion

What drives knowledge of word order during reading? Since the first report of flexibility in word position coding by Mirault et al. (2018), this has become a prominent debate. It has recently been established in several studies that readers' tendency to the ignore incorrectness of transposed adjacent words (e.g., 'the' and 'ignore' in this sentence) may persevere even when words are presented sequentially (Hossain & White, 2023; Huang & Staub, 2023; Liu et al., 2022; Milledge et al., 2023; Mirault et al., 2022; however, see Snell & Nogueira-Melo, 2024). This suggests that word position coding may unfold, at least partly, in memory.

The OB1-reader model's take on knowledge of word order is that the brain maintains a spatiotopic sentence-level representation in working memory (Snell et al., 2018a, 2018b). Activated words would be mapped onto locations in this spatiotopic representation via visual cues and top-down (grammatical) expectations. Although I and others have previously assumed that this process largely unfolds during perceptual stages where word recognition is still ongoing (e.g., Mirault et al., 2018; Snell & Grainger, 2019), the fact that the spatiotopic representation is rooted in working memory makes that the model is nonetheless easily harmonized with a scenario that emphasizes a role for memory over perception. But, at the same time, the model does not yet offer a mechanistic account of how words are bound to locations in the spatiotopic representation.

The present study was intended to kill two birds with a single stone: I sought to obtain further evidence for a role of the spatiotopic representation in working memory; and additionally, drawing inspiration from Feature Integration Theory (e.g., Treisman & Gelade, 1980), I sought to test a key role for visuo-spatial attention in the process of binding words to locations.

In line with prior investigations of word position coding flexibility, I let readers make grammaticality judgements, whereby the crucial comparison concerned the ease of rejecting transposed word (TW) sequences (e.g., *'the was dog here'*) versus ungrammatical control sequences (e.g., '*the man dog here*'). The novelty of the present experiment was the addition of a retro-cue, right after sentence offset, that appeared either at a critical location (where, prior to sentence offset, one could have locally established the incorrectness) or a non-critical location (where one could not have established the incorrectness). If TW effects were modulated by the location of these retro-cues, this would at once evidence a role for attention and a role for memory in word position coding.

In line with the hypotheses, readers had a significantly easier time detecting the incorrectness of TW sentences upon receiving valid retro-cues, expressed as a benefit of Δ ~ 22 ms compared to invalid retro-cues. Since sentences were replaced by post-masks when the retro-cue appeared, it is unlikely that the retro-cue affected processing in iconic (sensory) memory (see e.g., Agaogly et al., 2015 Smithson & Mollon, 2006); thus effects are taken to have unfolded in a subsequent working memory stage instead.

While the current results are in line with the hypothesis that focused attention binds words to locations in working memory, it is worth considering potential alternative explanations of these effects. For instance, could it be that focusing attention on two critical words merely led to better retention (i.e., prioritization) of these words in working memory, allowing for an easier response as compared to when two non-critical words were prioritized? This scenario does not involve the word position-coding process specifically. I reckon, however, that it is implausible: if the retro-cues simply drove prioritization generally rather than word position coding specifically, then the validity of the retro-cue should have impacted the ungrammatical control sequences just as much as the TW sequences. It can be seen in Fig. 2 (and from the statistically significant modulation of TW effects by cue validity) that this was not the case.

It is also worth considering the fact that effects in this study were quite subtle: modulations of TW effects by cue validity were expressed in RTs but not in accuracy, and cueing effects in RT were not substantial – at least when compared, for instance, with the main effect of sentence grammaticality (TW vs. ungrammatical control). Is this a reason for concern? My own view is that the cue itself was quite subtle: it consisted of an uppercase 'X' that was always surrounded by directly adjacent hashmarks from the post-mask (e.g., '*###X###*'). Thus, the impact of the cue may have been attenuated by crowding from the post-mask. Furthermore, the cue was only visible for 50 ms. Possibly its impact would have been stronger if it had been visible for a longer amount of time. It is important to note that the cue was designed to be this subtle on purpose: I did not want participants to consciously form task strategies based on the cues. And to a large extent this worked: out of the 54 participants retained for analysis, only eight reported having seen an occasional cue; and none of those eight participants could discern its purpose (indeed, cues were uninformative, with an equal number of occurrences between valid and invalid cues). In spite of the broad lack of awareness for these subtle cues, they had a significant impact. Further, the fact that cue validity only impacted RTs but not accuracy is, I believe, due to the fact that participants had to make speeded decisions in this experiment (unlike many traditional memory studies, which typically include waiting intervals prior to the response). I believe readers arrived at the same final percept in both the Valid and Invalid Cue trials. They merely arrived at that final percept a bit faster in the former than in the latter; hence the effect in RTs but not accuracy.

Looking forward, how might the notion of attention binding words to locations in working memory be consolidated further? Firstly, while I assume to have manipulated visuo-spatial attention with my retro-cues, it would of course help to have ways to track attention directly in order to verify this assumption. Although covert attention has long been rather elusive, the field has in recent years conceived of various methods to track it directly (e.g., Kornrumpf, Dimigen, & Sommer, 2017; Snell, Mathôt, Mirault, & Grainger, 2018). I particularly take note of recent developments with the so-called Rapid Invisible Frequency-Tagging (RIFT) technique, which combines eye-tracking with EEG to provide a direct measure of attention with high spatio-temporal resolution (e.g., Pan et al., 2021; Pan et al, 2023). The crux of RIFT is that various words are made to flicker at various rapid (and thus invisible) frequencies; and the extent to which coherence for any frequency can be decoded from the EEG signal reveals the amount of processing that occurred for the stimulus that flickered in that frequency. I believe this would be a very useful tool in the TW paradigm: if the four words are made to flicker at different frequencies, one could subsequently track the amount of attention that is allocated to each word; and the prediction would be that TW illusions would coincide with an attentional allocation to multiple words.

Another future perspective is to further investigate the timing of word position coding via manipulation of the timing of retro-cues. In the current study cues appeared immediately upon the disappearance of the sentence, meaning cues already appeared 200 ms after the start of word and sentence processing. If word position coding were to largely unfold in memory (rather than during perception), then possibly these cues were relatively early. If indeed later cues were to have a greater effect, then this would further underpin the post-perceptual nature of word position coding.

In conclusion, here I have investigated roles for attention and memory in word position coding. Bearing in mind the OB1-reader model (Snell et al., 2018a, 2018b) and the fact that the location of retro-cues modulated TW effects, I am compelled to posit that focused attention binds activated words to locations in a spatiotopic representation in working memory.

## Data Availability

All stimuli, software, data and analysis code are openly accessible via the Open Science Framework at https://osf.io/qx8c5/

## Footnotes

1 Indeed, the importance of word order is one of the reasons that some believe that readers process words in a strictly serial fashion (e.g., Reichle, Liversedge, Pollatsek, & Rayner, 2009).

2 Twenty sentences had a transposition of words 1 and 2; 42 sentences had a transposition of words 2 and 3; 18 sentences had a transposition of words 3 and 4.

3 Thirty-six sentences had a valid cue between words 1 and 2; 18 sentences had a valid cue between words 2 and 3; 26 sentences had a valid cue between words 3 and 4. Meanwhile, invalid cues occurred 26 times between words 1 and 2, 20 times between words 2 and 3, and 34 times between words 3 and 4.

4 R syntax: *DV* ~ *Grammaticality*Cue* + *(1* + *Grammaticality*Cue|Participant)* + *(1* + *Grammaticality*Cue|Item).*

## Code availability

All stimuli, software, data and analysis code are openly accessible via the Open Science Framework at https://osf.io/qx8c5/

## Declarations

### Open Practices statement

All stimuli, software, data and analysis code are openly accessible via the Open Science Framework at https://osf.io/qx8c5/
